# Ventricular and atrial function assessment with transthoracic echocardiography in patients with rheumatic inflammatory disease

**DOI:** 10.1186/s43044-022-00319-0

**Published:** 2022-11-25

**Authors:** Somayyeh Norouzi, Amirmohammad Khalaji, Mansoor Namazi, Somaye Sadat Rezaei, Amir Hossein Behnoush, Maryam Masoumi

**Affiliations:** 1 Department of Internal Medicine Science, Qom University of Medical Sciences, Qom, Iran; 2 School of Medicine, Tehran University of Medical Sciences, Tehran, Iran; 3 Cardiology Research Department, Qom University of Medical Sciences, Qom, Iran; 4 Faculty of Medicine, Qom University of Medical Sciences, Qom, Iran; 5 Clinical Research and Development Center, Shahid Beheshti Hospital, Qom University of Medical Sciences, Qom, Iran

**Keywords:** Rheumatic diseases, Systemic lupus erythematosus, Systemic scleroderma, Rheumatoid arthritis, Echocardiography

## Abstract

**Background:**

Inflammatory rheumatic diseases, including systemic lupus erythematosus (SLE), rheumatoid arthritis (RA), and systemic sclerosis (SSc), can cause cardiovascular complications in many cases. This study aimed to compare the ventricular and atrial functions of the heart between rheumatic patients and healthy controls using transthoracic echocardiography (TTE).

**Results:**

The study was performed between 64 patients with mentioned rheumatic diseases and 64 age- and sex-matched healthy controls who all underwent detailed history-taking and TTE. Echocardiographic parameters were measured and compared between the two groups. TTE showed significant differences in many echocardiographic parameters. Left ventricular end-diastolic diameter, left ventricular end-systolic diameter, right atrium area, inferior vena cava diameter, and systolic pulmonary artery pressure were significantly higher in patients compared to the controls (*P* < 0.001). Left ventricular ejection fraction and right ventricular end-diastolic diameter were not statistically different between the groups (*P* > 0.05). Right ventricular septal strain, right ventricular free wall strain, average longitudinal right ventricular strain, tricuspid annular plane systolic excursion, right ventricular systolic myocardial velocity, and right ventricular fractional area change were lower in inflammatory rheumatic patients (*P* < 0.001). The subgroup analysis showed the same results’ trend for each disease and its own control group comparison.

**Conclusions:**

Cardiac involvement in rheumatologic diseases, especially SLE, RA, and SSc, should always be taken into consideration as there may be silent changes affecting the overall prognosis of patients. Using TTE helps diagnose and make a treatment plan for cardiovascular complications in rheumatic disease patients.

## Background

Rheumatic diseases include but are not limited to systemic lupus erythematosus (SLE), rheumatoid arthritis (RA), vasculitis, systemic sclerosis (SSc), and spondyloarthropathies can cause several comorbidities, among which cardiovascular ones contribute the most to the mortality [1]. These manifestations have been identified and discussed extensively, as some may be the first presentation of the disease [2]. Rheumatic diseases can affect several aspects of the cardiovascular system, from the pericardium, myocardium, and heart valves to the cardiac conduction system and vasculature [3]. These mainly stem from a combination of traditional cardiovascular risk factors, such as age, gender, obesity, hypertension, diabetes mellitus and smoking, and inflammation caused by rheumatic diseases [4].

Transthoracic echocardiogram (TTE), the most common type of echocardiogram, is a non-invasive, available, and accurate modality to investigate the heart’s structure and diagnose any dysfunction in it. Left ventricular ejection fraction (LVEF), the most common representative of left ventricle (LV) systolic function is mainly measured by TTE. In addition, other parameters represent other cardiac functions and may be used in combination to find a certain pathology [5]. Cardiac manifestations of rheumatic diseases are described in the literature [6], however, few studies evaluated atrial and ventricular functions among patients with these diseases using echocardiography [7]. In this study, we aimed to compare the atrial and ventricular function of the heart between patients with inflammatory rheumatic diseases and age- and sex-matched healthy controls using TTE for the detection of subclinical changes in rheumatic patients.

## Methods

### Study design and subjects

In this case–control study, 64 patients with confirmed known inflammatory rheumatic diseases and 64 healthy individuals matched to the patients on the basis of age and sex referred to our institute were selected. All the patients selected were known cases of rheumatic inflammatory disease, including SSc, SLE, RA, or mixed connective tissue disease (MCTD) and none of them were in their flare episodes. All the controls were screened for rheumatic inflammatory diseases and were healthy. We excluded individuals with previous cardiac symptoms or known cardiovascular diseases and controls that used possibly confounding medication. Past medical history was taken from patients, including any chronic underlying diseases, smoking status, and drug history. This study was approved by the ethics committee of the Qom University of Medical Sciences (IR.MUQ.REC.1400.225) and informed consent was taken from all participants.

### Initial clinical assessment

An expert cardiologist measured the heart rate (HR), systolic blood pressure (SBP), and diastolic blood pressure (DBP) in both the patients and the control group. Height and weight were measured using common clinical devices by a research clinician. Body surface area (BSA) was reported according to the American Society of Echocardiography (ASE) as a commonly used metric for body size modification from weight and height using Boyd’s formula [8].

### Echocardiography assessment

TTE was performed according to standardized procedures for the patients and healthy individuals. The cardiologist interpreting echocardiograms was blinded to the group of participants. All procedure techniques and formulas were according to the ASE guidelines [8]. LVEF, LV end-diastolic diameter (LVEDD), LV end-systolic diameter (LVESD), right ventricular end-diastolic diameter (RVEDD), right atrium (RA) area, right ventricular (RV) septal strain, RV free wall strain, average longitudinal RV strain, tricuspid annular plane systolic excursion (TAPSE), RV systolic myocardial velocity, RV fractional area change (RVFAC), inferior vena cava (IVC) diameter, and systolic pulmonary artery pressure (SPAP) were reported by the sonographer. All measurements were also compared in each rheumatic disease group, including SLE, RA, and SSc.

### Statistical analysis

Continuous variables are presented as mean ± standard deviation (SD). The Student’s *t*-test was used to assess the significance of differences between inflammatory rheumatic patients and healthy individuals; and other analyses for continuous variables. The Chi-square test was used for categorical variables to calculate *P* value. Subgroup analyses were performed based on the rheumatic disease when possible. Statistical significance was considered a two-sided *P* value of less than 0.05 in analyses. All analyses were performed using the Statistical Package for the Social Sciences (SPSS), version 26 (IBM Corp., Armonk, NY, USA).

## Results

### Patient population and baseline characteristics

We included 64 patients with inflammatory rheumatic diseases comprised of SLE (*n* = 30), SSc (*n* = 21), RA (*n* = 10), and MCTD (*n* = 3) in addition to 64 age- and sex-matched healthy individuals. The duration of rheumatic diseases was 36 [12–75] (median [interquartile range]) months. Four patients with hypothyroidism, three with anemia, and two with mild fatty liver were recognized upon history-taking. Smoking was seen only in two patients. Each group consisted of 11 males and 53 females. There was no significant difference between patients and controls in terms of SBP (115.05 ± 6.20 vs. 111.55 ± 18.82; *P* = 0.160), DBP (73.45 ± 5.26 vs. 73.22 ± 4.52; *P* = 0.787), and BSA (1.661 ± 0.0572 vs. 1.664 ± 0.0497; *P* = 0.767). A significant difference between baseline HR was seen between patients and controls (79.61 ± 6.99 vs. 76.75 ± 6.84; *P* = 0.021). Table 1 summarizes the demographic data of participants.

**Table 1:** Baseline characteristics

	Cases				Controls (*n* = 64)	*p* value
	All rheumatic diseases (*n* = 64)	SLE (*n* = 30)	SSc (*n* = 21)	RA (*n* = 10)		
*Sex (n)*						
Male	11	6	2	2	11	1.000
Female	53	24	19	8	53	
Age	38.22 ± 11.09	35.13 ± 9.19	41.33 ± 13.79	42.10 ± 8.90	37.64 ± 9.57	0.753
Systolic blood pressure (mmHg)	115.05 ± 6.20	116.17 ± 5.67	113.52 ± 7.51	114.90 ± 5.45	111.55 ± 18.82	0.160
Diastolic blood pressure (mmHg)	73.45 ± 5.26	74.33 ± 5.25	72.52 ± 5.53	72.20 ± 4.73	73.22 ± 4.52	0.787
Heart rate (pulse/min)	79.61 ± 6.99	80.20 ± 8.77	78.52 ± 5.47	79.20 ± 4.18	76.75 ± 6.84	0.021
Body surface area (m^2^)	1.661 ± 0.0572	1.667 ± 0.0392	1.640 ± 0.0780	1.681 ± 0.0477	1.664 ± 0.0497	0.767

Data are represented as mean ± standard deviation for continuous variables

*SLE* systemic lupus erythematosus, *SSc* systemic sclerosis, *RA* rheumatoid arthritis

### Echocardiographic findings

The complete results of TTE are available in Table 2. There were no significant differences in LVEF and RVEDD between patients and controls (53.91 ± 6.00 vs. 55.16 ± 0.88, *P* = 0.104 and 28.66 ± 2.69 vs. 28.05 ± 1.69, *P* = 0.128, respectively). However, LVEDD, LVESD, and RA area were significantly higher in patients compared to the controls (*P* < 0.001). Moreover, there were significantly higher IVC diameter and PASP in patients compared to the controls (15.03 ± 3.18 vs. 12.06 ± 2.14, *P* < 0.001, and 30.61 ± 8.51 vs. 19.19 ± 1.92, *P* < 0.001, respectively). All other measures, including average longitudinal RV strain, TAPSE, RV systolic myocardial velocity, and RV fractional change area were significantly lower in patients compared to the controls (*P* < 0.001).

**Table 2:** Echocardiography results

	All rheumatic diseases			SLE (*n* = 30)			Systemic sclerosis (*n* = 21)			Rheumatoid arthritis (*n* = 10)		
	HC (*n* = 64)	Cases (*n* = 64)	*P* value	HC	SLE	*p* value	HC	SSc	*p* value	HC	RA	*p* value
LVEF (%)	55.16 ± 0.88	53.91 ± 6.00	0.104	55.17 ± 0.91	54.33 ± 2.54	0.099	55.24 ± 1.09	55.00 ± 2.24	0.663	55.00 ± 0.00	54.50 ± 1.58	0.331
LVEDD (mm)	40.03 ± 3.35	47.23 ± 3.82	< 0.001	39.57 ± 3.45	47.03 ± 4.13	< 0.001	39.48 ± 2.66	46.95 ± 3.58	< 0.001	41.90 ± 3.76	48.20 ± 3.05	0.001
LVESD (mm)	23.27 ± 4.84	30.86 ± 4.90	< 0.001	23.10 ± 4.55	30.37 ± 5.23	< 0.001	22.19 ± 4.66	29.52 ± 3.43	< 0.001	25.30 ± 5.62	33.50 ± 3.92	0.001
RVEDD (mm)	28.05 ± 1.69	28.66 ± 2.69	0.128	27.90 ± 1.71	28.37 ± 3.00	0.463	28.00 ± 1.55	28.38 ± 2.08	0.505	28.30 ± 2.06	29.00 ± 1.83	0.431
RA area (cm^2^)	11.41 ± 1.90	13.07 ± 2.85	< 0.001	11.50 ± 1.80	13.17 ± 3.42	0.022	11.62 ± 2.29	12.67 ± 2.35	0.152	11.10 ± 1.45	13.05 ± 2.17	0.029
RV septal strain (%)	21.05 ± 2.11	16.33 ± 4.00	< 0.001	20.83 ± 2.35	16.03 ± 4.31	< 0.001	20.67 ± 1.93	15.90 ± 2.93	< 0.001	22.00 ± 1.49	17.40 ± 4.95	0.017
RV free wall strain (%)	26.47 ± 2.59	23.23 ± 4.14	< 0.001	26.73 ± 1.87	23.07 ± 4.00	< 0.001	26.19 ± 1.89	22.57 ± 3.70	< 0.001	25.80 ± 4.76	24.30 ± 4.52	0.479
Average longitudinal RV strain (%)	24.09 ± 2.08	19.31 ± 3.68	< 0.001	24.10 ± 1.99	19.03 ± 3.52	< 0.001	23.48 ± 1.89	18.67 ± 3.10	< 0.001	24.80 ± 2.25	20.70 ± 4.57	0.020
TAPSE (mm)	25.61 ± 1.53	21.70 ± 2.99	< 0.001	25.77 ± 1.28	21.77 ± 2.49	< 0.001	25.62 ± 1.53	22.33 ± 2.94	< 0.001	25.10 ± 2.28	20.90 ± 3.32	0.004
RV systolic myocardial velocity (cm/s)	15.86 ± 1.48	12.73 ± 2.30	< 0.001	15.97 ± 1.40	12.70 ± 2.31	< 0.001	16.00 ± 1.34	13.05 ± 2.06	< 0.001	15.10 ± 2.02	12.50 ± 1.51	0.004
RV fractional area change (%)	37.83 ± 3.13	29.53 ± 7.18	< 0.001	37.67 ± 2.93	29.44 ± 7.37	< 0.001	37.14 ± 3.18	27.42 ± 6.41	< 0.001	38.80 ± 3.22	32.30 ± 4.42	0.001
IVC diameter (mm)	12.06 ± 2.14	15.03 ± 3.18	< 0.001	11.67 ± 2.12	15.07 ± 2.91	< 0.001	12.48 ± 2.46	15.52 ± 3.38	0.002	12.00 ± 1.33	13.30 ± 2.11	0.120
SPAP	19.19 ± 1.92	30.61 ± 8.51	< 0.001	19.17 ± 1.58	30.43 ± 9.84	< 0.001	18.90 ± 2.00	31.81 ± 7.42	< 0.001	20.20 ± 2.62	27.80 ± 5.10	0.001

Data are represented as mean ± standard deviation for continuous variables

*HC* healthy control, *SLE* systemic lupus erythematosus, *SSc* systemic sclerosis, *RA* rheumatoid arthritis, *LVEF* left ventricular ejection fraction, *LVEDD* left ventricular end-diastolic diameter, *LVESD* left ventricular end-systolic diameter, *RVEDD* right ventricular end-diastolic diameter, *RA* right atrium, *RV* right ventricle, *TAPSE* tricuspid annular plane systolic excursion, *IVC* inferior vena cava, *SPAP* systolic pulmonary artery pressure

In rheumatic disease subgroups, the results were almost similar with minor differences. There was no significant difference in the RA area between SSc patients and their controls (12.67 ± 2.35 vs. 11.62 ± 2.29, *P* = 0.152). In RA cases and controls, the RV-free wall strain and IVC diameter lost their significance (*P* = 0.479 and *P* = 0.120, respectively).

## Discussion

In this study, we evaluated the atrial and ventricular function of inflammatory rheumatic disease patients and compared them with healthy controls. Almost all parameters in patients with rheumatic disease except LVEF and RVEDD significantly differed from the control group. Subgroup analyses showed cardiac involvement in each SLE, SSc, and RA group.

Rheumatic diseases are responsible for myocardial, pericardial, valvular, electrical, and vascular changes in the cardiovascular system [3]. Increased levels of proinflammatory cytokines [9], atherosclerosis [10], chronic inflammation [11], and underlying autoimmune mechanisms are related to cardiovascular involvement in patients with rheumatic diseases [12]. As cardiovascular manifestations of rheumatic diseases may be silent or mild, early diagnosis and treatment help reduce mortality and morbidity [2]. Our findings implied that even rheumatic disease patients on treatment may have abnormal TTE findings.

SLE is an autoimmune disease that can affect various organs, including the cardiovascular system [13]. It has been shown that cardiovascular events are also higher in SLE patients compared with healthy controls [14]. In our study, LVEF was not significantly different between SLE patients and healthy individuals, which is similar to the study by Huang et al. [15], although, a difference was observed when it came to the 3D echocardiography in this study. While LVEDD and LVESD were higher in SLE patients, RVEDD and RA were the only indifferent parameters between SLE and healthy subjects. Luo et al. assessed echocardiographic findings in SLE patients with different levels of pulmonary hypertension and they found no difference in terms of RVEDD and RV fractional area curve and TAPSE except for the ones with moderate/severe pulmonary hypertension [16]. All in all, cardiac involvement is serious in SLE patients, and even in asymptomatic patients; a non-invasive method such as echocardiography can be helpful for the early detection of abnormalities [17].

Manifestations of SSc as another autoimmune disease are not limited to the skin, in a way that it can affect the whole body, including musculoskeletal, pulmonary, gastrointestinal, renal, endocrine, and cardiovascular systems [18]. Cardiac complications of SSc may manifest as myocardial or pericardial damage, conduction system fibrosis, and valvular diseases; however, pulmonary hypertension caused by SSc is also responsible for cardiovascular involvement [19]. In a study by Huez et al., right ventricular diastolic dysfunction was seen among patients diagnosed with SSc, caused by latent pulmonary hypertension [20]. Following with the mentioned study, our study showed that the SPAP in the patient group was approximately 1.7 times that of the control group, indicating the presence of pulmonary hypertension in a significant proportion of SSc patients. Therefore, a simple TTE can have clinical implications in preventing this serious complication.

RA with a prevalence of about 1%, can have extra-articular presentations, including cardiovascular ones [21, 22]. Pericarditis is the most common cardiac manifestation, followed by myocarditis, congestive heart failure, cardiomyopathy, and pericardial effusion [23]. The findings of our study suggest left ventricular dysfunction in RA cases shown by several parameters. This is in line with the results of a study by Myasoedova et al., which showed abnormal LV remodeling in RA patients without heart failure [24].

Although we did our best to make the study flawless, there were some limitations to our study. First, all the patients were selected from one center with the same ethnicity, which may threaten its generalizability. Second, despite highly-skilled cardiologists performing the TTE, this test is operator-related and may be erroneous in some cases. Finally, echocardiographic assessment in inflammatory rheumatic diseases has not been done much in the literature, and further research with more sample size is highly recommended.

## Conclusions

In conclusion, cardiac involvement in rheumatic diseases, especially SLE, SSc, and RA should always be considered as there may be silent changes in the heart affecting the overall prognosis of patients. Echocardiography is a safe and feasible tool for clinicians to diagnose and prevent such complications and reduce the total burden of the diseases.

## Data Availability

The datasets generated and/or analyzed during the current study are not publicly available due they are part of a database, containing further, not yet published data. However, data used in this study are available from the corresponding author on reasonable request.

## Abbreviations

SLE: Systemic lupus erythematosus
RA: Rheumatoid arthritis
SSc: Systemic sclerosis
TTE: Transthoracic echocardiography
LVEF: Left ventricular ejection fraction
LV: Left ventricle
MCTD: Mixed connective tissue disease
HR: Heart rate
SBP: Systolic blood pressure
DBP: Diastolic blood pressure
BSA: Body surface area
ASE: American Society of Echocardiography
LVEDD: Left-ventricular end-diastolic diameter
LVESD: Left-ventricular end-systolic diameter
RVEDD: Right-ventricular end-diastolic diameter
RV: Right ventricular
TAPSE: Tricuspid annular plane systolic excursion
RVFAC: Right ventricular fractional area change
IVC: Inferior vena cava
SPAP: Systolic pulmonary artery pressure
SD: Standard deviation
SPSS: Statistical package for the social sciences
